# The association between longitudinal changes in inter-hemispheric IPS functional connectivity and math gains depends on children’s age and task requirements

**DOI:** 10.1016/j.dcn.2025.101666

**Published:** 2025-12-30

**Authors:** Macarena Suárez-Pellicioni, Gavin Price, James R. Booth

**Affiliations:** a Department of Educational Studies in Psychology, Research Methodology, and Counseling, University of Alabama, AL, USA; b Department of Psychology, University of Exeter, Exeter, United Kingdom; c Department of Psychology and Human Development, Vanderbilt University, TN, USA

**Keywords:** Hemispheric, Parietal, Functional connectivity, Age, Longitudinal, Math, Children

## Abstract

The role of the approximate number system (ANS) in scaffolding symbolic mathematics remains unresolved. A prior neuroimaging study from our group (Suárez-Pellicioni & Booth, 2018) found no significant longitudinal effects of ANS acuity—indexed by intraparietal sulcus (IPS) activation—on gains in math fluency. However, the absence of age-specific analyses and exclusive focus on fluency, which emphasizes retrieval, may have contributed to these null findings. To address these limitations, the present study examined whether age moderates the relationship between inter-hemispheric IPS functional connectivity during a non-symbolic comparison task and math skill. Specifically, we tested: (1) baseline associations at Time 1 (T1); (2) whether T1 connectivity predicts gains in math skill over time (scaffolding hypothesis); and (3) whether changes in connectivity relate to longitudinal gains. Forty-eight children completed a dot comparison task in the scanner at T1 and again two years later. Standardized measures of subtraction skill and math fluency were collected at both time points. We measured general psychophysiological interaction (gPPI) between IPS seeds and contralateral IPS regions. For subtraction skill, we found no evidence of a concurrent association at T1 or predictive effects of T1 connectivity moderated by age. However, changes in connectivity over time revealed an age-dependent pattern: younger children showed gains linked to increased right-left parietal connectivity, while older children showed gains with decreased connectivity. This suggests a developmental shift from effortful integration to more efficient processing. Effects were specific to subtraction, not fluency.

## 1 Introduction

When you glance at two checkout lines in a store and quickly choose the shorter one, you are likely relying on an evolutionarily ancient ability to estimate and compare numerical quantities without counting. This ability, that we share with nonhuman primates, birds, and amphibians, is known as the Approximate Number System (ANS; (Dehaene et al., 1999, Feigenson et al., 2004). Representations within the ANS are inherently noisy and imprecise, show meaningful individual differences, and tend to become more refined across development (Halberda et al., 2012). Numerous studies have demonstrated that the acuity of non-symbolic numerical representations in the ANS is associated with performance in the uniquely human ability for symbolic mathematics, such as doing arithmetic equations like 5 + 8 = 13 (Chen and Li, 2014; De Smedt et al., 2013; Fazio et al., 2014; Schneider et al., 2017). However, the directionality of this relationship remains unclear and is the focus of ongoing investigation. One possibility, known as the scaffolding (or mapping) hypothesis, suggests that this innate sense of quantity serves as a foundational mechanism for developing more abstract, symbolic mathematical understanding. An alternative view, known as the refinement hypothesis, proposes that experience with symbolic number manipulation, a cultural invention, enhances the neural representation of quantity within the ANS.

Longitudinal studies are essential to clarify between these two hypotheses. Several longitudinal studies have supported the scaffolding hypothesis (Desoete et al., 2012, Praet et al., 2013, Sasanguie et al., 2012; Soto-Calvo et al., 2015). For example, infants’ ANS was found to predict math skill at 3.5 years of age (Starr et al., 2013), preschoolers’ ANS precision predicted performance on school math at 6 years of age (Mazzocco et al., 2011) even when accounting for initial differences in math skill (Libertus et al., 2013). Further support for the scaffolding hypothesis comes from training studies showing that improving ANS acuity leads to gains in symbolic math skill in both preschoolers (Wang et al., 2021) and adults (Park and Brannon, 2013), including when modality-independent ANS representations are targeted (Libertus et al., 2020). Evidence challenging the scaffolding hypothesis comes from correlational studies finding only weak associations between ANS and symbolic math achievement (Caviola et al., 2020) and from meta-analyses of ANS training studies finding no significant improvement in symbolic math skill following ANS training (Qiu et al., 2020; Szucs and Myers, 2017).

Longitudinal studies employing cross-lagged panel designs (Kearney, 2003) have found evidence supporting the refinement hypothesis. For example, Mussolin, Nys, Content, and Leybaert (2014) found that symbolic math skill in 3- to 4-year-olds, but not ANS acuity, predicted symbolic math skill seven months later. Similarly, Lau et al. (2021) reported that symbolic number ability was the strongest predictor of subsequent ANS acuity in kindergarteners. Additional longitudinal evidence comes from Matejko and Ansari (2016), who examined developmental trajectories and found that symbolic math at the beginning of first grade, but not ANS, predicted later symbolic math skill, whereas both early symbolic math and ANS acuity predicted later ANS acuity. These findings support the refinement hypothesis and challenge the scaffolding role of ANS. Examining individuals with varying levels of exposure to formal mathematics, (Nys et al., 2013), found that Western adults without formal math instruction showed less precise ANS acuity than those who had received mathematical education. Research on the Amazonian Mundurucú population similarly showed that adults who had studied math exhibited greater ANS precision than those who had not, even when controlling for age (Piazza et al., 2013). Moreover, Lindskog et al. (2014) found that ANS acuity improved as a function of study duration among business university students. Overall, these results support the refinement hypothesis. (For a longitudinal study finding evidence for bidirectional associations between ANS precision and symbolic math skill over time in 3-to-5-year-old children, see Elliott et al., 2019).

To our knowledge, only one study has directly investigated the scaffolding and refinement hypotheses using functional Magnetic Resonance Imaging (fMRI; Suárez-Pellicioni and Booth, 2018). In this study, children completed a non-symbolic dot comparison task inside the scanner at approximately 10 years of age and again two years later. Symbolic math skill was assessed at both timepoints using a standardized measure of math fluency. ANS acuity was indexed by brain activation during the dot comparison task within the bilateral intraparietal sulcus (IPS), which is situated between the inferior and superior parietal lobules (IPL/SPL). This region is considered the core neural substrate for quantity representation (Dehaene et al., 2003), as it exhibits ratio-dependent activation: greater neural response to difficult ratios (i.e., numerically close quantities) compared to easier ones (Pinel et al., 2001). This study employed a cross-lagged panel approach to examine the temporal influence of IPS activation on symbolic math gains (scaffolding hypothesis) and the influence of initial symbolic math skill on changes in IPS activation over time (refinement hypothesis). Our findings revealed that higher symbolic math skill at Time 1 predicted increased IPS activation at Time 2, even after controlling for initial IPS activation. Crucially, we found no evidence supporting the scaffolding hypothesis: ANS-related IPS activation did not predict subsequent improvement in math fluency. This study provided the first neuroimaging evidence in favor of the refinement hypothesis, suggesting that experience with symbolic number manipulation, a uniquely human, culturally constructed skill, can refine an ancient cognitive system that we share with other species and offering a compelling illustration of how education and experience shape the brain.

One limitation of our study was its focus on brain activation within the IPS. While this region is central to quantity representation, complex skills such as mathematical learning are increasingly understood to rely on large-scale brain networks, which may be better captured by analyses of functional connectivity rather than activation alone (Bressler and Menon, 2010). Psychophysiological interaction (PPI) analysis provides a method for assessing task-dependent connectivity between brain regions. High correlations in activation suggest that regions are co-activated and functionally interacting during task performance.

Although previous studies have linked parietal connectivity to concurrent and prospective math skills, their designs do not allow for direct conclusions regarding the scaffolding hypothesis. Park et al. (2013) used PPI analysis to identify significant functional connectivity between a number-selective seed in the right IPS and the left parietal cortex during a subtraction task in adults. They found that inter-hemispheric parietal connectivity correlated with response times in subtraction, but not addition. However, they did not examine gains over time, limiting causal inference about the role of ANS in scaffolding math skill. Moreover, their seed regions were defined using a conjunction of contrast maps from a number matching task and two non-symbolic arithmetic tasks, with requirements extending beyond ANS-specific processing and encompassing broader parietal areas (e.g., hIP3 and superior parietal lobule). In a longitudinal study, Price et al. (2018) reported that greater resting-state functional connectivity between right IPS subdivisions (hIP1 and hIP2) and left IPS (hIP3) at the end of first grade predicted better math skill at the end of second grade. However, because this was a resting-state study, it did not directly measure ANS-related processing, and math skill was not measured at Time 1, an omission that complicates causal inference (Merkley et al., 2017). Finally, Battista et al. (2018) examined children aged 7–14 and found that children with higher math skill maintained consistently strong IPS connectivity across timepoints. However, their seed region (hIP3) was anatomically defined without a functional task to localize quantity representation, and the stability of age-normed math scores across timepoints precluded analysis of gains in symbolic math skill. Taken together, existing literature has not directly examined the role of functional connectivity between non-symbolic processing regions in bilateral IPS in predicting the development of symbolic math skills over time.

Our previous neuroimaging study (Suárez-Pellicioni et al., 2018) had two limitations in addressing the scaffolding hypothesis. First, the absence of evidence supporting the scaffolding hypothesis may have been influenced by the age of participants at Time 1, who spanned a relatively wide age range (8.4–14.1 years), potentially obscuring age-dependent effects. It is possible that ANS acuity serves a scaffolding role in younger children but not in older ones, as suggested by prior research (Cai et al., 2018). Therefore, we might have found support for the scaffolding hypothesis had we studied younger children or explicitly examined the role of age. Second, the role of ANS in scaffolding symbolic math may depend on the specific type of symbolic math (i.e. operation) being assessed. Several investigations have demonstrated distinct cognitive and neural demands for different arithmetic operations (Suárez-Pellicioni et al., 2022). Subtraction tends to rely on parietal-based quantity representation and calculation strategies (Suárez-Pellicioni et al., 2020, Suárez-Pellicioni et al., 2021), whereas multiplication is more strongly associated with temporo-frontal retrieval-based mechanisms (Suárez-Pellicioni et al., 2019). It is therefore possible that our 2018 study did not find evidence for the scaffolding hypothesis because math skill was assessed using a timed math fluency test, in which participants solved as many single-digit arithmetic problems as possible within three minutes. Different results might have emerged had we assessed math with an untimed subtraction test, which relies more heavily on IPS-based quantity processing.

The present study aimed to address these limitations by employing general psychophysiological interaction (gPPI; McLaren et al., 2012) analyses to investigate three key questions. First: Does age moderate the relationship between inter-hemispheric parietal functional connectivity during a non-symbolic dot comparison task at Time 1 and math skill as measured by subtraction skill or math fluency at Time 1 (concurrent associations)? Although the focus on Time 1 of this question does not allow us to test the scaffolding hypothesis, this analysis provides useful baseline information into the neural correlates of mathematical skill. Second: Does age moderate whether inter-hemispheric parietal connectivity at T1 predict gains over time in subtraction skill or math fluency (brain as a predictor)? Answering this question was critical for evaluating the scaffolding hypothesis, as it would provide evidence that interhemispheric parietal functional connectivity between ANS regions serves as a *precursor* or *causal* factor in explaining longitudinal gains in math skill. Third: Does age moderate whether changes over time in inter-hemispheric parietal connectivity is associated with gains in subtraction skill or math fluency (T2–T1; changes in brain and behavior)? While this question does not allow us to directly test the scaffolding hypothesis, it offers valuable insights into the sustained role of ANS-related connectivity in relation to development of math skill. For all three questions: 1) inter-hemispheric connectivity was defined as the connectivity between a seed defined in the right IPS and an ROI defined in left IPS (hereafter, right-left), and between a seed in the left IPS and an ROI defined in right IPS (hereafter, left-right); 2) Age was treated as a continuous variable, and follow-up analyses for younger and older children subgroups were conducted only when a significant interaction with age was observed; 3) Both subtraction skill and math fluency was studied, with the effects expected for the former but not for the latter (Suárez-Pellicioni et al., 2022).

## 2 Materials and methods

### 2.1 Participants

#### 2.1.1 Whole sample

Sixty-five children were recruited from schools in the Chicago metropolitan area to participate in the study. All participants were native English speakers and right-handed. They were free of past and present neurological or psychiatric disorders and had no history of attention deficits or oral language deficits, according to parental reports. Participants had no intellectual deficits, all of them scoring above 85 standard scores (SS) on intelligence as measured with the Wechsler Abbreviated Scale of Intelligence – WASI (Weschler, 1999). Reading deficits were ruled out by including in the study those participants who scored above 80 SS on the average of the Word Attack and Word Identification subtests of the Woodcock-Johnson III Test of Achievement (WJ-III; Woodcock et al., 2001).

Data from 17 participants were excluded from the analysis for the following reasons: 1) Excessive movement inside the scanner, that is, more than 10 % of the total volumes replaced (i.e. 11 volumes) or more than five consecutive volumes replaced in a single run (T1: n = 1, T2: n = 4). ArtRepair was used to identify volumes with significant movement (see section 2.7.1. for more details). 2) Low accuracy on the dot comparison task solved inside the scanner (n = 7), defined as accuracy being below 50 % in any of the conditions either at Time 1 or at Time 2. All the excluded participants had low accuracy on the control condition at both timepoints and on all the numerosity conditions at T2. 3) Reading deficit, defined as 80 SS on the average of Word Attack and Word Identification subtests of the WJ-III (n = 1). 4) Missing data for one of the runs of the dot comparison task, due to lack of time (n = 1). 5) Problem with the program used to present stimuli (Eprime; n = 1). 6) Lack of subtraction skill measure at T2 to calculate longitudinal gains (n = 1). 7) Bad coverage of frontal/parietal cortices at T2 (n = 1).

The final sample consisted of 48 participants who were approximately 11 years old at T1 and approximately 13 at T2.^1^ Table 1 shows information about the final sample. Participants were scanned twice while they completed a dot comparison task inside the scanner, sessions being approximately 2.5 years apart from one another. Subtraction skill and math fluency were measured at each time point to calculate gains over time (T2 minus T1).

**Table 1:** Sample information. Sex ratio and means (standard deviation in parenthesis) for age at T1 and T2, time between sessions, and standardized tests (subtraction skill, math fluency, reading skill, verbal and visuospatial working memory, and intelligence) for the whole sample (n = 48) and for the younger (n = 24) and older (n = 24) groups.

	Whole sample	Younger	Older
Age at T1^(1)^	11.1 (1.4)	10.0 (0.6)	12.3 (0.9)
Age at T2^(1)^	13.5 (1.5)	12.3 (0.6)	14.7 (1.0)
Time between sessions^(1)^	2.3 (0.3)	2.3 (0.2)	2.4 (0.3)
Female/male ratio	25/23	15/9	10/14
Standardized tests			
Subtraction T1^(2)^	14.4 (5.0)	13.0 (5.1)	16.1 (4.3)
Subtraction T2^(2)^	16.4 (4.4)	16.0 (5.2)	16.8 (3.6)
Subtraction gains T2-T1^(2)^	2.0 (3.8)	3.2 (3.7)	0.9 (3.6)
Math fluency T1^(2)^	62.1 (24.8)	54.8 (22.2)	69.4 (25.5)
Math fluency T2^(2)^	82.4 (27.6)	75.8 (25.8)	89.1 (27.6)
Math fluency gains T2-T1^(2)^	20.3 (15.8)	21.0 (17.3)	20.0 (14.1)
Reading skill at T1^(3)^	106.6 (11.0)	107.2 (10.7)	106.0 (11.1)
Verbal WM^(3)^	102.5 (13.4)	101.5 (11.3)	103.5 (15.6)
Visuospatial WM^(3)^	105.2 (13.2)	108.3 (12.2)	102.0 (13.6)
Full IQ at T1^(3)^	111.4 (15.3)	114.9 (15.2)	108.0 (15.0)

Note. ^(1)^ in years. ^(2)^ raw scores. ^(3)^ standardized scores.

#### 2.1.2 Ethics statement

Written assent and consent was obtained from the children and their parents or guardians, respectively. All experimental procedures were approved by the Institutional Review Board at Northwestern University and participants were compensated $20 per hour for their time.

#### 2.1.3 Age groups

**Fig. 1:**
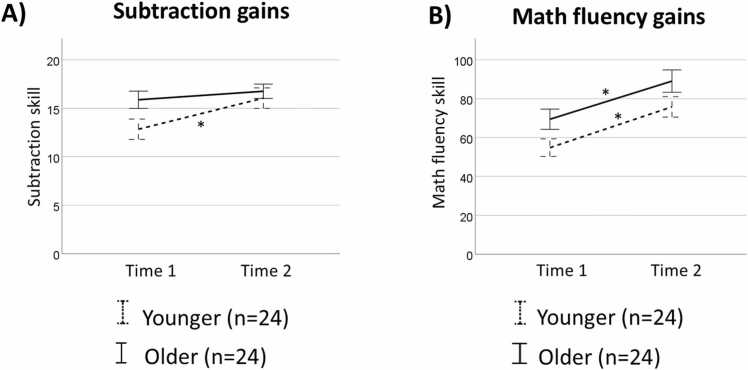
Gains in subtraction skill and math fluency separately for each age group. Gains over time in (A) subtraction skill and (B) math fluency for younger (dotted line) and older (full line) children. Error bars represent the standard error of the mean. **Note.**^(*)^*p* = .001.

While age was treated as a continuous variable in all interaction analyses, we categorized participants into two groups (younger and older) for follow-up analyses when a significant interaction between age and skill/improvement was observed (see section 2.7.6, Second Level Analysis). The younger group comprised 24 participants who were approximately 10 years old at T1 (age range: 8.47–10.94) while the older group comprised 24 participants who were approximately 12 years old at T1 (age range: 10.97–14.06). More information about these groups is given in Table 1. Gains in subtraction and math fluency over time, separately for each age group, are shown in Fig. 1. The two groups differed in age at T1 (*t*(46) = -10.34, *p* < .001, *partial η²* = .70), age at T2 (*t*(46) = -9.77, *p* < .001, *partial η²* = .68) (as expected), subtraction skill at T1 (*t*(46) = -2.21, *p* = .03, *partial η²* = .10), gains in subtraction skill (*t*(46) = 2.21, *p* = .03, *partial η²* = .10), and math fluency at T1 (*t*(46) = -2.12, *p* = .04, *partial η²* = .09). The two groups did not differ in time between sessions (*t*(46) = -1.30, *p* = .20, *partial η²* = .04), reading skill (*t*(46) = .38, *p* = .71, *partial η²* = .003), subtraction skill at T2 (*t*(46) = -.55, *p* = .59, *partial η²* = .006), math fluency at T2 (*t*(46) = -1.70, *p* = .10, *partial η²* = .06), gains in math fluency (*t*(46) = .30, *p* = .77, *partial η²* = .002), verbal (*t*(45) = -.51, *p* = .61, *partial η²* = .006) or visuospatial (*t*(45) = 1.69, *p* = .10, *partial η²* = .06) working memory (WM), full IQ (*t*(46) = 1.57, *p* = .12, *partial η²* = .05) or sex distribution (*X*^*2*^ = 2.09, *p* = .15).

### 2.2 Standardized measures

#### 2.2.1 Math gains measures

**Longitudinal gains in subtraction skill:** The subtraction subtest of the Comprehensive Mathematical Abilities Test (CMAT; Hresko et al., 2003) was used to measure gains in subtraction skill. This is an untimed test including 23 subtraction problems that are solved in paper-and-pencil format. It has a wide range of difficulty, including very simple single-digit subtractions, multi-digit subtractions, subtraction of decimals, and subtraction of fractions. The tests were measured both at T1 and at T2. Longitudinal gains in subtraction skill were defined as the changes in raw scores over time (T2-T1).

**Longitudinal gains in math fluency:** Math fluency was measured with the Math Fluency subtest of the WJ-III (Woodcock et al., 2001), which requires the rapid calculation of single-digit addition, subtraction, and multiplication facts within a 3-minute time limit. The test includes 160 problems. This test was administered at both time points and longitudinal gains in math fluency were defined as the changes in raw scores over time (T2-T1).

#### 2.2.2 Control measures

**Reading ability:** Reading skill was measured as the average of standardized scores on the Word Attack and the Word Identification subtest from the Woodcock-Johnson III Test of Achievement (WJ-III; (Woodcock et al., 2001) at T1. The Word Attack requires reading phonically regular nonsense words while the Word Identification test requires reading isolated letters and real words.

**Working memory:** Verbal working memory (WM) was measured by the Listening Recall subtests of the Automated Working Memory Assessment (AWMA; (Alloway et al., 2007). This subtest requires participants to decide whether a sentence is true or false (e.g. *“Bananas live in water”*,) and to remember the final word of the sentence (e.g. “*water*”). Participants are asked to store the final word of the sentence, as they process an increasing number of new sentences. Items are scored as correct if participants recall the correct word or words in the correct order.

Visuospatial WM was measured with the Spatial Recall subtest of the AWMA (Alloway et al., 2007). In this test, participants view pictures of two shapes where the shape on the right has a red dot near it and they need to identify whether the shape on the right is the same as the shape on the left when rotated in two dimensions, or whether it is the mirror image. At the end of each trial, individuals are asked to remember the position of the red dot and to answer by pointing to a picture with three possible positions marked. The number of shape pairs to be compared increases as participants proceed through the test, and they must recall the correct position of all the red dots in the correct temporal order.

**Intelligence**: Both performance and verbal IQ were measured with the Wechsler Abbreviated Scale of Intelligence – WASI (Weschler, 1999). Performance IQ was measured with Block Design and Matrix Reasoning subtests of the WASI. The Block Design test requires the participants to use red-and-white blocks to re-create the model design shown by the examiner, within a specified time limit. In the Matrix Reasoning subtest, the examinee views an incomplete matrix or series and selects the response option that completes it. Verbal IQ was measured with the Vocabulary and Similarities subtests of the WASI. In the Vocabulary subtest, participants have to define words, while in the Similarities one participants are presented two words that represent common objects or concepts and they have to describe how they are similar.

### 2.3 Task solved inside the scanner: dot comparison task

**Fig. 2:**
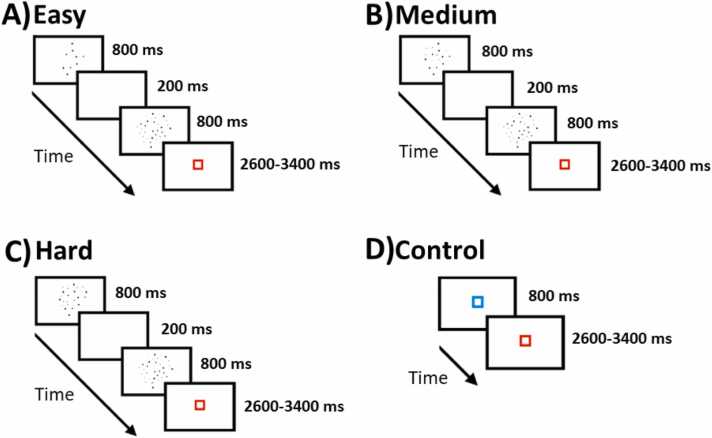
Dot comparison task solved inside the scanner and its timing. Dot comparison task including (A) Easy ratio requiring the comparison of 12 vs. 36 dots; (B) Medium ratio requiring the comparison of 18 vs. 36 dots; (C) Hard ratio requiring the comparison of 24 vs. 36 dots. (D) Control condition in which participants had to respond with their index finger when the blue squared turned into red.

Participants were sequentially presented with two dot arrays and their task was to decide which of them had more dots. The task comprised 24 easy (i.e. compare 12 vs. 36 dots; Fig. 2A), 24 medium (i.e. 18 vs. 36; Fig. 2B), and 24 hard (i.e. 24 vs 36 dots; Fig. 2C) trials. The first dot array was composed of the larger number of dots in half of the trials, while it was composed of the smaller number of dots in the other half. To ensure that participants’ judgments were based on differences in quantity rather than cumulative surface area, the distribution of dot sizes was biased towards smaller dots in large arrays and bigger dots in small arrays. However, totally equating the cumulative surface area between small and large arrays by entirely biasing the distribution of single dot sizes (100 % bias) may have led participants to use single dot sizes as a cue for their judgments. Therefore, we found a trade-off (50 % bias) between equating as much as possible the cumulative surface areas and the distributions of single dot sizes in each pair. Additionally, we included 24 control trials in order to control for basic attention and motor responses. Control trials consisted of a blue square that was presented for the same duration as the experimental conditions (i.e. 3 ratios) and participants were asked to press a button when the square turned red (Fig. 2D). The total number of trials was 96, which were divided into two separate runs with 48 trials each. Twelve trials of each condition were presented in the practice session, which were different from the ones used in the scanning sessions.

### 2.4 Experimental protocol

First, consent was obtained, and standardized tests were administered. Then, participants completed a practice session in which they practiced the task and learned to minimize head movement in a mock fMRI scanner. The actual scanning session took place within a week of the practice session. In the fMRI scanner, participants performed two runs of the dot comparison task. Stimuli were projected onto a screen that was viewed by the participants through a mirror attached to the head-coil. The timing and order of trial presentation within each run was optimized for estimation efficiency using optseq2 (http://surfer.nmr.mgh.harvard.edu/optseq/). Behavioral responses were recorded using an MR-compatible keypad and participants responded with their right hand. Participants responded with their index finger if the first array of dots was composed of more dots and with the middle finger if the second array of dots was composed of more dots. For the control condition, participants responded with their index finger when the blue squared turned into red.

### 2.5 Stimulus timing

A trial started with the presentation of the first set of dots for 800 ms, followed by a blank screen for 200 ms. The second set of dots was then presented for 800 ms, followed by a red fixation square for 200 ms. Variable periods of fixation (ranging from 2600 ms to 3400 ms; 400 ms jitter) were added after each trial, during which the red square was presented. Furthermore, each run ended with 22 s of passive visual fixation in order to aid in deconvolution of the final trials.

### 2.6 fMRI data acquisition

Images were collected using a Siemens 3 T TIM Trio MRI scanner (Siemens Healthcare, Erlangen, Germany) at CAMRI, Northwestern University’s Center for Advanced MRI. Before functional image acquisition, a high-resolution T1 weighted 3D structural image was acquired for each subject, with the following parameters: TR = 1570 ms, TE = 3.36 ms, matrix size = 256 × 256, field of view = 240 mm, slice thickness = 1 mm, number of slices = 160. The fMRI blood oxygenation level dependent (BOLD) signal was measured with a susceptibility weighted single-shot echo planar imaging (EPI) sequence. The following parameters were used: TE = 20 ms, flip angle = 80 s, matrix size = 128 × 120, field of view = 220 × 206.25 mm, slice thickness = 3 mm (.48 mm gap), number of slices = 32, TR = 2000 ms.

### 2.7 fMRI data analysis

#### 2.7.1 Preprocessing

Data analysis was performed using SPM12 (www.fil.ion.ucl.ac.uk/spm). The first six images of the run were discarded to allow for T1 equilibration effects. The remaining functional images were corrected for slice acquisition delays and realigned to their mean functional image across runs. The anatomical image was then segmented and warped to the default tissue probability map (TPM) template to get the transformation field. An anatomical brain mask was created by combining the segmentation products (i.e. grey matter, white matter, and cerebrospinal fluid) and then applied to its original anatomical image to produce a skull-stripped anatomical image. After that, the mean functional image and all functional images were coregistered to the skull-stripped anatomical image. Images were then normalized to the standard T1 Montreal Neurological Institute (MNI) template and spatially smoothed with a Gaussian filter equal to about twice the voxel size (4 × 4 × 8 mm^3^ full width at half maximum). To reduce movement effects on brain signal, Art-Repair (https://cibsr.stanford.edu/tools/human-brain-project/artrepair-software.html; (Mazaika et al., 2009) was used to identify outlier volumes, defined as those with volume-to-volume head movement exceeding 1.5 mm in any direction, head movement greater than 5 mm in any direction from the mean functional image across runs, or deviations of more than 4 % from the mean global signal intensity. The outlier volumes were repaired by interpolation between the nearest non-outlier volumes. Subjects included in our study had no more than 10 % of the volumes repaired in each run and no more than 5 consecutive volumes repaired in each run. Six motion parameters estimated in the realignment step were entered in the first-level modeling as regressors and the repaired volumes were deweighted (Mazaika et al., 2009).

### 2.8 fMRI processing: first level

Event-related statistical analysis was performed according to the general linear model (GLM). Activation was modeled as epochs with onsets time-locked to the presentation of the first stimulus in each trial (i.e. first set of dots). The whole duration of the trial was modeled. All epochs were convolved with a canonical hemodynamic response function. The time series data were high-pass filtered (1/128 Hz), and serial correlations were corrected using an autoregressive AR model.

#### 2.8.1 Functional connectivity analysis

Functional connectivity analysis was carried out using a general psychophysiological interaction analysis (gPPI; http://www.nitrc.org/projects/gppi) implemented in SPM 12. This analysis allows an investigation of the connectivity strength from one brain area to other areas under a certain experimental condition (McLaren et al., 2012). This gPPI method consistently estimates psychophysiological interactions with greater accuracy than the standard implementation of PPI in SPM 8 (McLaren et al., 2012). By modeling all the conditions independently instead of simply subtracting two conditions, gPPI reduced both false positives and false negatives, particularly in experiments involving more than two conditions (McLaren et al., 2012).

The data was modeled at the individual level by including two runs of the dot comparison task at T1 and two runs at T2, including the following regressors into a GLM model: the four experimental parameter regressors (easy, medium, and hard ratios, and control condition), the four psychophysiological interaction (PPI) regressors of the interaction, the time series from the seed region and the six motion regressors of head movement. The four experimental parameters regressors were formed using the onset times for the easy, medium, and hard ratios and control condition of the dot comparison task. In total, the model included 60 regressors (4 + 4 + 1 + 6 ×2 runs x 2 time points).

#### 2.8.2 Seeds definition

**Fig. 3:**
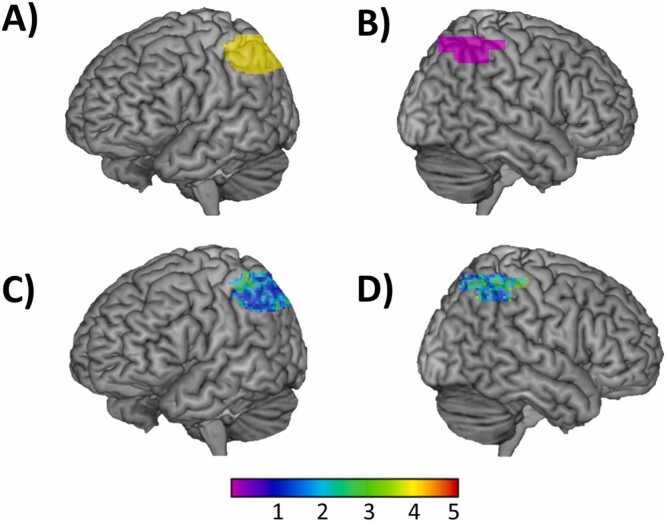
Anatomical regions used to individually define the seeds and as regions of interest (ROIs). The anatomically defined (A) left and (B) right IPS were used as the regions of interest and to individually extract the seed for every participant. Seed overlap across participants in the anatomical left (C) and right (D) IPS. The color bar shows the number of participants showing overlap at every given voxel. Greater overlap is shown in green.

Seeds were defined individually. Two seeds were defined separately for each participant. Seeds were defined as the top 50 voxels showing maximum activation strength for the contrast “hard greater than easy” of the dot comparison task at T1 within the left and right intraparietal sulcus (IPS), respectively. This was an a priori decision. IPS was defined anatomically, by first dilating (3D dilation of 2) the inferior and superior parietal lobules (IPL/SPL) and then getting the intersection between the two (Suárez-Pellicioni and Booth, 2018a). The IPL and SPL were defined anatomically using the automated anatomical labeling (AAL) template, which is part of the Wfupickatlas tool. Fig. 3 shows the (A) left IPS and (B) right IPS from which the seeds were individually defined. The same seeds were used to answer the three questions of this study. Fig. 3 shows seed overlap across participants in the (C) left IPS and (D) right IPS.

#### 2.8.3 ROIs definition

**Fig. 4:**
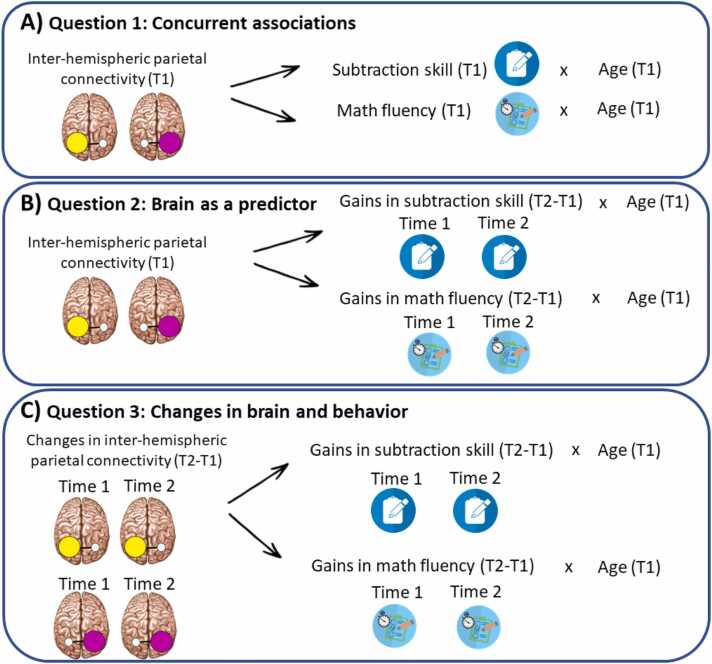
Illustration of the main variables used in the regression analyses carried out to answer the three research questions of this study. Illustration of the main variables used in the regression analyses to address: (A) Question 1 (concurrent associations): This question aimed to address the association between inter-hemispheric parietal connectivity at T1 and the interaction between age and math skill (subtraction skill and math fluency) at T1. (B) Question 2 (brain as a predictor): This question aimed to address the association between inter-hemispheric parietal connectivity at T1 and the interaction between age and gains in math skill (subtraction skill and math fluency) over time. (C) Question 3 (changes in brain and behavior): This question aimed to address the association between changes in inter-hemispheric parietal connectivity (increases and decreases) and the interaction between age and math skill (subtraction skill and math fluency) gains over time. For all three questions, inter-hemispheric parietal connectivity refers to the connectivity between the right IPS seed and the left IPS ROI (i.e. right-left) as well as the connectivity between the left IPS seed and the right IPS ROI (i.e. left-right).

ROIs were defined anatomically, in the bilateral IPS. IPS was defined as described above, by first dilating (3D dilation of 2) the inferior and superior parietal lobules (IPL/SPL) and then getting the intersection between the two (Suárez-Pellicioni and Booth, 2018). The same ROIs were used to answer the 3 research questions of this study (see Fig. 4). This decision was exploratory. Previous exploration included defining the ROIs as a combination between the brain activation elicited by the “hard vs. easy” contrast of the numerosity task within the anatomical left and right IPL/SPL and the “all ratios vs. control” contrast of the numerosity task within the anatomical left and right IPL/SPL. Fig. 3 shows the ROIs in the (A) left IPS, in yellow, and in the (B) right IPS, in violet.

Statistical significance for these ROIs was determined using Monte Carlo simulations in AFNI’s 3dClustSim program (December 2015; see http://afni.nimh.nih.gov/, with SPM’s data smoothness parameters, autocorrelation function = 0.47, 4.65, 10.56). A cluster size of 29 and 25 voxels was needed to reach significance for the left IPS and right IPS ROIs, respectively. Clusters exceeding these thresholds, at a cluster-wise threshold of *p* = .05 and voxel-wise threshold of *p* = .005, were deemed significant.

#### 2.8.4 s-level analysis

To answer the three research questions of this study, we followed the same approach of previous publications of this group (e.g. Suárez-Pellicioni, Demir-Lira, et al., 2021; Suárez-Pellicioni et al., 2018b, 2019b), by running cluster-wise regression analyses.

Question 1 **Concurrent associations**

This question aimed to address the role of age in explaining the association between inter-hemispheric parietal connectivity and math skill at T1 (Fig. 4A). Four regression analyses were carried out to answer this question. The first regression included the interaction between age and subtraction skill at T1 as the covariate of interest, with the main effects of age and subtraction skill at T1, and full IQ^2^ as the covariates of no interest. The second regression included the interaction between age and math fluency at T1 as the covariates of interest, with the main effects of age and math fluency at T1, and full IQ as the covariates of no interest. Both regressions were carried out for the seeds defined in left and right IPS. All variables were continuous measures. The contrast of interest in this analysis was the comparison between ratios at time 1 (hard > easy at T1).

Question 2 **Brain as a predictor.**

This question aimed to address the role of age in explaining the association between inter-hemispheric parietal connectivity and longitudinal gain in math skill (Fig. 4B). Four regression analyses were carried out to answer this question. The first regression analysis included the interaction between age and subtraction gains as the covariate of interest, with the main effects of age and subtraction gains, and full IQ as the covariates of no interest. Then, we carried out another regression analysis including the interaction between age and math fluency gains as the covariate of interest, with the main effects of age and fluency gains, and full IQ as the covariates of no interest. Both regressions were carried out for the two seeds. All variables were continuous measures. The contrast of interest in this analysis was the comparison between ratios at time 1 (“hard > easy at T1”).

Question 3 **Changes in brain and behavior**

This question aimed to address the role of age in explaining the association between changes in inter-hemispheric parietal connectivity over time and longitudinal gains in math skill (Fig. 4C). To answer this question, we used the regression analyses carried out to answer Question 2, but instead of looking at brain activation at T1, the contrasts of interest were changes in brain connectivity over time for the contrast comparing the ratios: “hard > easy T1 > T2” and “hard > easy T2 > T1”.

**Follow-up analyses**

For all the questions, follow-up analyses were carried out separately for younger and older children (see Section 2.1.3 for more details on age groups) when an interaction with age was found to be significant. For Question 1, follow-up analyses examined the association between connectivity at T1 and math skill at T1 separately for younger and older children, while controlling for full IQ. For Question 2, follow-up analyses explored the relationship between connectivity at T1 and math gains (T2–T1) separately for younger and older children, while controlling for full IQ. For Question 3, follow-up analyses investigated the association between changes in connectivity over time (T2–T1) and math gains (T2–T1), again separately for younger and older children, while accounting for full IQ.

Across all the analyses, we used the left IPS (Fig. 3A) to study the connectivity patterns emerging from the right IPS seed and we used the right IPS (Fig. 3B) to study the connectivity patterns emerging from the left IPS seed.

## 3 Results

### 3.1 Behavioral results: performance on the dot comparison task solved inside the scanner

We carried out four regression analyses including Ratio (easy, medium, hard) as the within-subjects factor, for percentage of accuracy and response times, both at T1 and at T2. The analysis revealed a significant effect of ratio for accuracy (T1: *F*(2,94) = 10.94, *p* < .001, *partial η²* = .19; T2: *F*(2,94) = 13.00, *p* < .001, *partial η²* = .22) and response times (T1: *F*(2,94) = 17.34, *p* < .001, *partial η²* = .27; T2: *F*(2,94) = 17.62, *p* < .001, *partial η²* = .27). As expected, participants were faster and more accurate responding to easy than harder ratios. While addressing the brain correlates of task performance is not the aim of this study, these findings suggest that the task successfully elicited the intended effects by engaging quantity mechanisms.

### 3.2 fMRI results

We present the results for the three research questions for subtraction first, followed by math fluency.

#### 3.2.1 No concurrent associations (Question 1)

The regression analyses to answer Question 1 revealed no cluster showing a significant association between inter-hemispheric parietal connectivity at T1 and the interaction between age and subtraction skill at T1 for the whole sample, regardless of the hemisphere of the seed.

#### 3.2.2 Lack of reliable effects for the role of brain as a predictor (Question 2)

The analyses to answer Question 2 revealed that connectivity between the right IPS seed and a cluster in the left IPS showed a significant interaction between age and subtraction gains. However, this interaction was no longer significant when three outliers identified via Cook’s Distance were removed from the fMRI analysis. This raises concerns about the stability of the association and suggests that individual variability may have disproportionately influenced this result.

#### 3.2.3 Subtraction skill gains are associated with increases in right-left parietal connectivity over time among younger children (Question 3)

**Fig. 5:**
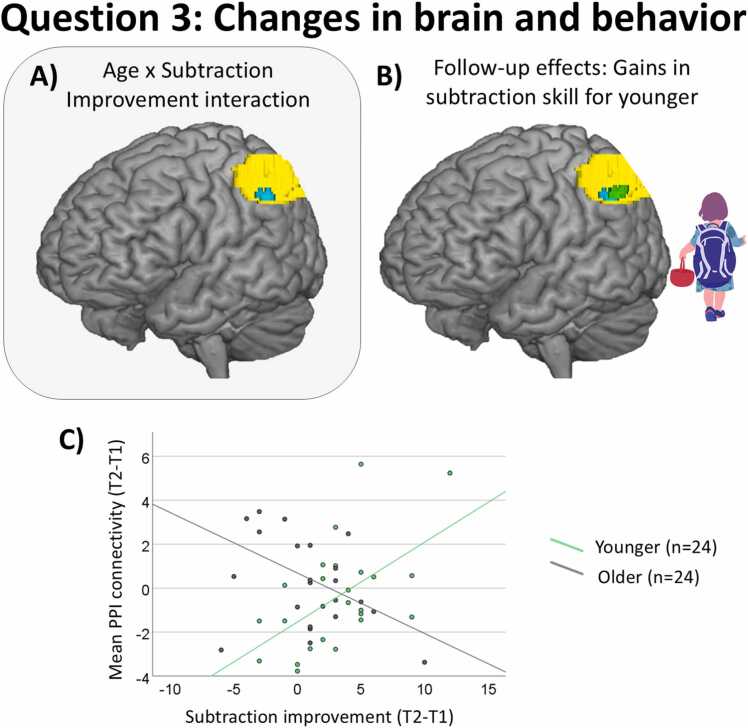
Changes in right-left parietal connectivity over time associated with subtraction gains in younger children (Question 3). (A) Cluster in left IPS (in cyan) whose changes in connectivity over time with the right IPS seed was associated with the interaction between age and subtraction gains for the whole sample. (B) Cluster in left IPS (in green) whose changes in connectivity over time with the right IPS seed was associated with longitudinal gains in subtraction skill for younger children. This cluster overlays the cluster showing an age by subtraction gains interaction (in cyan) and the ROI in the left IPS (in yellow). (C) Scatterplot showing a positive association between changes in right-left parietal connectivity over time and subtraction gains for younger children (in green) and a negative association for older children (in dark gray). Error bars represent the standard error of the mean.

The analyses to answer Question 3 revealed that connectivity between the right IPS seed and a cluster in the left IPS showed an interaction between age and subtraction skill gains. This cluster is shown in cyan in Fig. 5A. Note that this cluster continued to be significant: 1) once three outliers identified via Cook’s Distance were removed; 2) if the seed was defined as top 100 or top 200 voxels showing activation for the hard vs. easy contrast of the dot comparison task, instead of the top 50 voxels (which led to greater seed overlap across participants), or if the seed is defined in the whole anatomical right IPS; 3) if subtraction gains adjusted for baseline differences at T1 (i.e., residuals) was used in the analysis, instead of raw change (T2-T1). Overall, these additional analyses show the robustness of this finding.

**Fig. 6:**
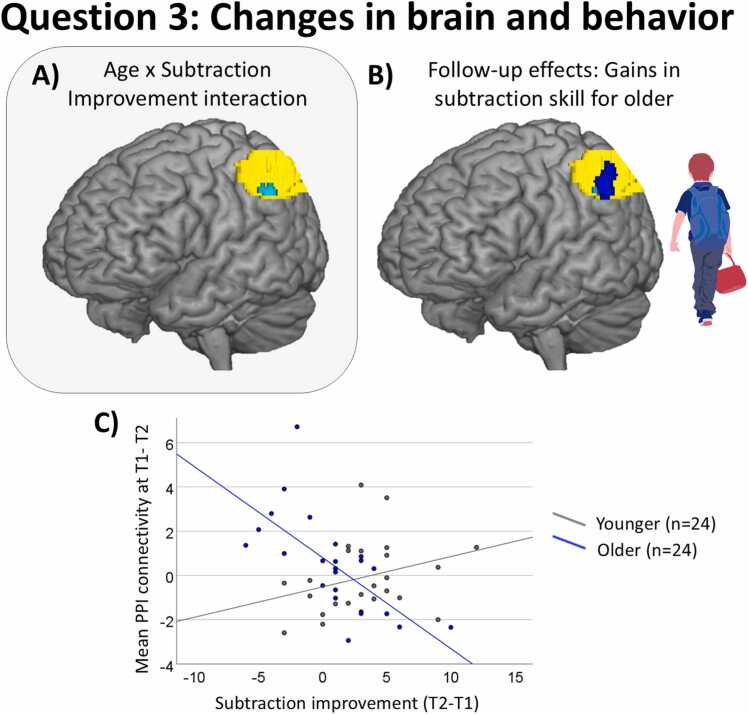
Changes in right-left parietal connectivity over time associated with subtraction gains in older children. (A) Cluster in left IPS (in cyan) whose changes in connectivity over time with the right IPS seed was associated with the interaction between age and subtraction gains for the whole sample. (B) Cluster in the left parietal cortex (in blue) whose changes in connectivity over time (T1 > T2) with the right IPS seed was associated with longitudinal gains in subtraction skill for older children. This cluster overlays the cluster showing the age by subtraction gains interaction (in cyan) and the ROI in the left IPS (in yellow). (C) Scatterplot showing a negative association between changes in right-left parietal connectivity over time (T1 > T2) and subtraction gains for older children (in blue) and a positive trend for younger children (in dark gray). Error bars represent the standard error of the mean.

**Fig. 7:**
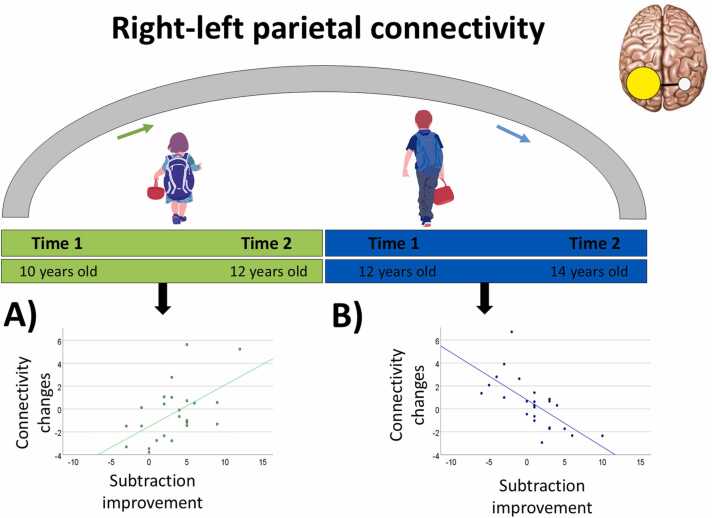
Summary of connectivity results. (A) Right-left parietal connectivity increases over time in association with longitudinal gains in subtraction skill for younger children (Question 3; in green). (B) Right-left parietal connectivity decreases over time in association with longitudinal gains in subtraction skill for older children (Question 3; in blue).

Follow-up analyses were calculated, looking at the association between subtraction gains and changes in connectivity separately for younger and older children. (For more information about these age groups, see Section 2.1.3.) More specifically, these analyses included longitudinal gains in subtraction as the covariate of interest and full IQ as the covariate of no interest. These analyses revealed that changes in connectivity between the right IPS seed and a cluster in the left IPS explained subtraction gains in younger children. This cluster is plotted in green in Fig. 5B, overlapping the cluster showing the age by subtraction gains interaction (shown in cyan) and the left IPS ROI (shown in yellow). More information about these clusters is given in Table 2. As shown in Fig. 5C, the higher the increases (T2 > T1 contrast) in right-left connectivity, the more younger children improved in subtraction skill over time, whereas the opposite pattern was observed for older children. This result is also shown in Fig. 7A, where all connectivity findings are summarized.

**Table 2:** Clusters showing a significant right-left parietal connectivity for Question 3. Approximate Brodmann areas (∼BA), cluster size (k), MNI coordinates of the peaks (x, y, z), and Z-values for the cluster showing a significant association between changes in right-left parietal connectivity over time and the interaction between age and subtraction gains for the whole sample (n = 48), and follow-up clusters showing a significant association between right-left connectivity changes and subtraction gains separately for younger (n = 24) and older children (n = 24).

			MNI coordinates				
Region	∼BA	k	X	Y	Z	Z value	P^a^
**Age x Improvement interaction for the whole sample (n = 48)**							
Left IPS (cluster in cyan in Fig. 5A & 6 A)	7/40	48	-20	-64	40	3.53	< .001
**Follow-up effects for younger children (n = 24)**							
Left IPS (cluster in green in Fig. 5B)	7/40	40	-36	-56	42	3.28	.001
**Follow-up effects for older children (n = 24)**							
Left IPS (cluster in blue in Fig. 6B)	7/40	168	-26	-58	46	4.08	< .001
			-20	-64	42	3.86	< .001
			-30	-62	54	3.21	.001

a Uncorrected p value of the peak.

No cluster reached significance for the opposite contrast (T1 > T2) or when the seed was defined in the left IPS for younger children.

#### 3.2.4 Subtraction skill gains are associated with decreases in right-left parietal connectivity over time among older children (Question 3)

As described above, a cluster showed a significant interaction between age and subtraction gains (in cyan in Figs. 5A and 6A). Besides the increases in connectivity associated with gains for younger children described above, follow-up analyses also revealed that changes in connectivity between the right IPS seed and a cluster in the left IPS were associated with subtraction gains in older children.^3^ This cluster is plotted in blue in Fig. 6B, overlapping the cluster showing the age by subtraction gains interaction (shown in cyan) and the left IPS ROI (shown in yellow). More information about these clusters is given in Table 2. As shown in Fig. 6C, the higher the decreases (T1 > T2 contrast) in right-left parietal connectivity, the more older children improved in subtraction skill over time whereas the opposite pattern was observed for younger children.

Although older children did not show significant changes in subtraction scores over time as a group (n = 24), there were notable individual differences in these changes within this group. This fMRI result remained consistent for a subsample of older children showing significant subtraction gains over time.

This result is also shown in Fig. 7B, where all connectivity findings are summarized.

No cluster reached significance for the opposite contrast (T2 > T1) or when the seed was defined in the left IPS for older children.

#### 3.2.5 Lack of effects for math fluency

No cluster emerged as significant when studying the role of inter-hemispheric parietal connectivity at T1 in association with the interaction between age and math fluency at T1 (Question 1) or math fluency gains (Question 2). No cluster reached significance when looking at changes over time in this connectivity in association with the interaction between age and longitudinal math fluency gains (Question 3). This was true regardless of where the seed was defined (right or left IPS).

## 4 Discussion

The role of the approximate number system (ANS) in scaffolding symbolic mathematics remains unresolved. While some behavioral studies support this hypothesis (Libertus et al., 2013, Libertus et al., 2020, Mazzocco et al., 2011, Starr et al., 2013, Wang et al., 2021), others report findings that contradict it (Caviola et al., 2020, Lau et al., 2021, Matejko and Ansari, 2016, Mussolin et al., 2014, Qiu et al., 2020; Szucs and Myers, 2017). A previous neuroimaging study from our group examined the directional relation between ANS acuity and symbolic math skills, revealing clear evidence that math skill at Time 1 (T1) refined later quantity representations in the parietal cortex. This longitudinal effect was observed in children who were approximately 10 years old at T1 and 12 years old at Time 2 (T2). However, we found no evidence that ANS acuity at T1 predicted later math skill, so there was no support for the scaffolding hypothesis.

Two methodological factors may account for the lack of support for the scaffolding hypothesis. First, the sample at T1 spanned a relatively wide age range (8.4–14.1 years), potentially obscuring age-dependent effects. Second, symbolic math was assessed using a timed math fluency test, which emphasizes retrieval rather than quantity-based processing. The present study addresses these limitations by investigating whether age moderates the association between ANS acuity and math skill, with the hypothesis that scaffolding effects may be more prominent in younger children (Cai et al., 2018). Additionally, we included an untimed measure of subtraction skill alongside math fluency, anticipating that subtraction, an operation more reliant on quantity processing, would be more sensitive to scaffolding effects than fluency-based measures.

To capture the dynamic neural mechanisms underlying numerical cognition (Moeller et al., 2015), we focused on functional connectivity analyses using psychophysiological interaction (PPI) methods. Specifically, we examined the degree of “communication” between the left and right intraparietal sulcus (IPS), rather than regional activation (Suárez-Pellicioni and Booth, 2018). Connectivity measures may be particularly sensitive to developmental and individual differences, revealing maturational changes that are not evident in activation patterns alone (Bressler and Menon, 2010).

This study addressed three research questions. First: Does age moderate the relation between inter-hemispheric parietal functional connectivity during non-symbolic dot comparison task at Time 1 and math skill as measured by subtraction skill or math fluency at Time 1 (concurrent associations)? Although the focus on Time 1 of this question does not allow us to test the scaffolding hypothesis, this analysis provides useful baseline information into the neural correlates of mathematical skill. Second: Does age moderate whether inter-hemispheric parietal connectivity at T1 predict gains over time in subtraction skill or math fluency (brain as a predictor)? Evidence of such predictive associations would support the scaffolding hypothesis. Third: Does age moderate whether changes over time in inter-hemispheric parietal connectivity is associated with gains in subtraction skill or math fluency over time (changes in brain and behavior)? While this question does not allow us to directly test the scaffolding hypothesis, it offers valuable insights into the sustained role of ANS-related connectivity in relation to development of math skill.

To address these questions, 48 students completed a dot comparison task in the scanner at both T1 and T2, approximately two years apart. Inter-hemispheric parietal functional connectivity was defined as the extent to which the left IPS (seed) interacted with the right IPS region of interest (ROI), and vice versa, during dot comparison trials involving hard versus easy numerical ratios. Participants also completed timed math fluency and untimed subtraction tasks at both timepoints, with score differences used to quantify gains in math skill. Age was treated as a continuous variable in all analyses, and follow-up analyses separately for younger and older groups were conducted only when significant age interactions were observed. The following sections present the results for each of the three research questions.

### 4.1 Lack of concurrent associations (Question 1)

Our analyses revealed that inter-hemispheric parietal connectivity was not concurrently associated with the interaction between age and math skill (i.e. subtraction skill or math fluency) at T1. It is difficult to interpret null findings, as they may reflect limited statistical power rather than a true absence of association. In our study, the lack of a significant association between age and math skill with brain connectivity at T1 should not be taken as definitive evidence of no association. Instead, it suggests that, within the constraints of our sample size and design, no reliable link was detected.

### 4.2 Lack of robust evidence supporting the scaffolding hypothesis (Question 2)

Our initial analyses revealed that right-left IPS connectivity at T1 was associated with the interaction between age and gains in subtraction skill; however, this effect did not remain significant after excluding outliers using Cook’s distance method. As a result, we were unable to identify a cluster whose connectivity reliably predicted longitudinal gains in subtraction skill as a function of age. This suggests that the association may have been disproportionately driven by a small number of participants with atypical connectivity or behavioral profiles. Although our analysis revealed no reliable result supporting the scaffolding role of ANS in the development of math skill, it could still be the case that the lack of robust findings can be attributed to low statistical power due to our small sample size. Our sample size of 48 participants presents a limitation of this study, particularly for individual differences analyses, where larger samples have been emphasized as crucial (Marek et al., 2022).

### 4.3 Gains in subtraction skills is associated with increasing right-left parietal connectivity over time for younger children (Question 3)

Our analysis to answer Question 3 revealed a cluster showing that the relation of longitudinal connectivity with gains in subtraction skill depended on age. Follow-up analyses separately for younger and older children revealed that younger children who improved in subtraction skill increased right-left parietal connectivity over time (Fig. 5, Fig. 7). This finding could be interpreted as younger children showing increasing reliance over time on the effortful integration of non-symbolic quantity representations (ANS) across bilateral IPS over time to improve their subtraction skills, consistent with the scaffolding hypothesis. However, it is also possible that increased connectivity is a consequence of gains in subtraction skill, which would align with the refinement hypothesis. Therefore, this result does not allow us to disentangle these two hypotheses, so we can only conclude that, at early stages of development, gains in subtraction skill are associated with greater functional connectivity between ANS-related regions in bilateral IPS.

### 4.4 Gains in subtraction skill is associated with decreasing right-left parietal connectivity over time for older children (Question 3)

We found that longitudinal gains in subtraction skill in older children was associated with decreased right-left connectivity (Fig. 6, Fig. 7). This contrasts with younger children who showed that gains were associated with increasing connectivity, suggesting non-linear relation as a function of development. This pattern suggests a developmental shift from effortful interhemispheric integration in younger children toward more efficient processing as subtraction becomes automatized in older children. This interpretation aligns with the behavioral trajectories observed across age groups: although younger children began with lower subtraction performance, both groups reached comparable levels by Time 2. This convergence implies that younger children probably were in a phase of active knowledge acquisition, whereas older children were likely consolidating existing knowledge, refining strategies, or improving processing efficiency. Such developmental differences may also explain why older children, as a group, did not exhibit significant changes in subtraction accuracy over time.

One possible interpretation of the finding of decreasing connectivity with increasing skill is that it reflects neural efficiency. The parietal cortex undergoes important anatomical changes in early adolescence, including a reduction in cortical gray matter density and synaptic pruning (Chechik et al., 1998). This pruning may streamline neural networks, making them more efficient and specialized for specific functions. In addition to these anatomical changes, the effect of experience and education on the brain (Suárez-Pellicioni and Booth, 2018) may have also played a critical role in reducing this connectivity around this age. From 12–14 years old, older children who show gains in math skill may have had enough experience with numerical processing that allows them to improve in subtraction while also reducing the “communication” needed between right and left IPS. Another possible interpretation for this decreased connectivity associated with gains in math skill for the older group is the estrangement hypothesis (Lyons et al., 2012). This hypothesis posits that symbolic and non-symbolic representations become increasingly disconnected as children get older and are exposed to symbolic numbers in formal educational settings. The detachment of the symbolic representations from their non-symbolic numeric referents could explain the reduced communication between ANS regions in bilateral IPS. This is consistent with a recent study finding age-related differences in the overlap between symbolic and non-symbolic representations in childhood and their association with arithmetic problem-solving skills (Schwartz et al., 2021). Younger children (7–10 years old) exhibited a positive correlation between arithmetic skill and cross-format neural representational similarity in the parietal cortex, but this association was absent in adolescents and young adults (14–21 years old). The authors concluded that mapping between symbolic and non-symbolic representations plays a crucial role in arithmetic processing early on, but reliance on this mapping decreases as fluency with symbolic numbers improves—consistent with the estrangement hypothesis (Lyons et al., 2012). Similarly, Bulthé et al. (2018) found that greater overlap between symbolic and non-symbolic representations was associated with poorer arithmetic skills in adults. Our finding of increased connectivity in younger children (10 years old) and decreased connectivity in older children (12 years old) —suggest enhanced mapping in younger children and estrangement in older children. However, we cannot definitively draw this conclusion, as our connectivity analysis was limited to a non-symbolic task.

### 4.5 Findings are specific for subtraction skill

We observed effects for subtraction skill, as measured by the CMAT but not for math fluency skill, as measured with the WJ-III. While the WJ-III test included subtraction, it differed from the CMAT in several ways. The CMAT encompassed a wide range of difficulty levels, likely more robustly engaging procedural strategies, whereas the WJ-III test focused on single-digit addition, subtraction, and multiplication facts, likely requiring a mix of retrieval and procedural strategies. In addition, the CMAT test was untimed, whereas the WJ-III test imposed a three-minute time limit. Overall, the emphasis of each test differed, with the CMAT primarily assessing problem-solving in more complex arithmetic tasks that engage quantity mechanisms, whereas the WJ-III test mainly measured children's automaticity in solving simple arithmetic problems.

The fact that all our findings are exclusive to gains in subtraction skill and not replicated for gains in math fluency is consistent with previous studies showing that different operations involve different brain mechanisms (Suárez-Pellicioni et al., 2022). Subtraction has been shown to be more reliant on parietal-based quantity-based mechanisms (Suárez-Pellicioni et al., 2020). Park et al., (2013) found inter-hemispheric parietal connectivity to explain individual differences in performance for subtraction, but not for addition. In contrast, math fluency relies more on automatic retrieval of arithmetic facts from memory and therefore engages frontal (Jost et al., 2011) and temporal (Prado et al., 2011, 2014; Sokolowski et al., 2022; Suárez-Pellicioni et al., 2019a) cortices to a greater degree.

### 4.6 Findings are specific for when the seed is defined in the right IPS

Studies analyzing parietal functional connectivity have defined the seed in either the right IPS (Park et al., 2013) or left IPS (Battista et al., 2018), but not bilaterally. Our study investigated inter-hemispheric connectivity after assessing both right-left (i.e. seed in right IPS; ROI in left IPS) and left-right (i.e. seed in left IPS; ROI in right IPS) inter-hemispheric connectivity (see also Price et al., 2018). Across all three research questions, our analysis revealed no significant effect for the left-right IPS connectivity. We believe this is because we defined the seed using a non-symbolic task (i.e. dot comparison task). Previous studies have suggested this task places greater demands on the right than on the left parietal cortex (Sokolowski et al., 2017). Future studies should address the role of right-left and left-right inter-hemispheric parietal connectivity during a symbolic comparison task (Lynn et al., 2022) in explaining gains in math skill (Park et al., 2014). Future studies should also address the role of the different IPS subdivisions in predicting math gains, as these have been suggested to show different patterns of functional and structural connectivity (Uddin et al., 2010) and to be differently associated with later math skill (Price et al., 2018).

### 4.7 Conclusions

This study investigated the role of the approximate number system (ANS) in math by analyzing inter-hemispheric parietal functional connectivity during a non-symbolic dot comparison task. We examined whether the relation of connectivity to math skill differed depending on age with the first and across two time points. Math skill was measured by subtraction skill, which places heavier demands on quantity-based mechanisms, and math fluency, which emphasizes automaticity of retrieval. Our findings revealed no significant associations between intra-parietal sulcus (IPS) connectivity at Time 1 (T1) and the interaction between age and math skill at T1 (Question 1) or gains in math skill from T1 to T2 (Question 2). As such, we found no robust evidence that early ANS-related connectivity facilitates later symbolic math development depending on age (i.e. no support for the scaffolding hypothesis). However, the absence of significant effects should be interpreted cautiously, as null results may reflect limited statistical power due to modest sample size.

Analyses addressing Question 3 revealed an age-dependent pattern in connectivity changes over time associated with gains in subtraction skill. Among younger children, gains in subtraction skill was linked to increased inter-hemispheric parietal connectivity over time, whereas older children showed gains in skill in association with decreased connectivity. This pattern suggests a developmental shift. Younger children may rely on more effortful inter-hemispheric integration during early stages of subtraction learning, whereas older children may engage in more efficient processing as the skill becomes automatized.

No significant associations were found for gains in math fluency, underscoring the specificity of these effects to skill measures that engage quantity-based processing, like subtraction (Suárez-Pellicioni et al., 2022).

Overall, the observed inverted U-shaped relationship between changes in inter-hemispheric connectivity and subtraction gains depending on age highlights the value of longitudinal fMRI approaches for understanding how neural communication evolves to support skill acquisition across development. Our results also highlight the importance of considering age not merely as a covariate, but as a moderator of the neural pathways underlying cognitive gains.

### CRediT authorship contribution statement

**James R. Booth:** Writing – review & editing, Resources, Methodology, Investigation, Funding acquisition. **Suárez Pellicioni Macarena:** Writing – review & editing, Writing – original draft, Visualization, Formal analysis, Data curation, Conceptualization. **Gavin Price:** Writing – review & editing.

## Funding

This research was supported by HD059177 from the 10.13039/100000071National Institute of Child Health and Human Development to JRB.

## Declaration of Generative AI and AI-assisted technologies in the writing process

During the preparation of this work the author(s) used Bing and Grammarly to brush up the English language after writing to enhance understandability and accessibility. After using this tool/service, the author(s) reviewed and edited the content as needed and take(s) full responsibility for the content of the published article.

## Declaration of Competing Interest

The authors declare that they have no known competing financial interests or personal relationships that could have appeared to influence the work reported in this paper.

## Data Availability

Data availability statement provided. The dataset used in this study is available in OpenNeuro (10.18112/openneuro.ds001486.v1.1.0).

## Footnotes

1 We selected this age group because the data is part of a larger dataset designed to investigate the neurocognitive mechanisms underlying multiplication and subtraction. To ensure participants had experience with both operations, we collected data from children who had been taught these skills in school, which typically occurs around third grade in the United States.

2 Full IQ was included as a covariate of no interest in the analyses given that it positively correlated with subtraction performance at both time points.

3 We conducted an exploratory analysis by excluding from the analysis 3 older participants showing the largest decrease in subtraction scores over time, which resulted in a sample of 21 participants showing significant improvement in subtraction over time (*t*(20) = -2.59, *p* = .02). We then repeated the fMRI analysis for this subsample (n = 21) and found a cluster in the left IPS (k = 237) that overlapped with the cluster obtained for the entire older sample (n = 24; k = 168)
