# Supplementary material for: The association between longitudinal changes in inter-hemispheric IPS functional connectivity and math gains depends on children’s age and task requirements
Source: Dev Cogn Neurosci. 2025 Dec 30;78:101666. doi: 10.1016/j.dcn.2025.101666 (PMC12813480; doi:10.1016/j.dcn.2025.101666)
Supplement: Supplementary material [file mmc1.docx]

**Supplementary materials**

- 1. **Age groups’ math performance**

To explore age-related differences in subtraction skill, we calculated a mixed ANOVA including Time (subtraction score at T1 and T2) as the within-subjects factor and Age groups (younger, older) as the between-subjects factor. This analysis showed a non-significant main effect of Age group (*F*(1,46) = 2.35, *p* = .13, *partial* *η²* = .05), a significant main effect of Time (*F*(1, 46) = 14.92, *p* < .001, *partial* *η²* = .25), and a significant Time x Age groups interaction (*F*(1,46) = 4.9, *p* = .03, *partial* *η²* = .10). The interaction showed that the two age groups differed in subtraction skill at T1 (*t*(46) = -2.21, *p* = .03, *partial* *η²* = .10), but not at T2 (*t*(46) = -.55, *p* = .59, *partial* *η²* = .006) and that the younger group showed significant changes over time (*t*(23) = -4.27, *p* < .001, *partial* *η²* = .44) in subtraction skill whereas the older one did not (*t*(24) = -1.18, *p* = .25, *partial* *η²* = .06). More detailed information about the groups’ subtraction skill at each time point is provided in Table 1 and Figure 1A.

We calculated the same ANOVA described above but including math fluency scores at T1 and T2 as the within-subjects factor (i.e. Time). This analysis revealed a main effect of Age groups (*F*(1,46) = 4.00, *p* = .05, *partial* *η²* = .08) and Time (*F*(1,46) = 78.2, *p* < .001, *partial* *η²* = .002), but not a significant Time x Age groups interaction (*F*(1,46) = .09, *p* = .76, *partial* *η²* = .002). The main effect of Time showed that both younger (*t*(23) = -6.0, *p* < .001, *partial* *η²* = .61) and older (*t*(23) = -6.66, *p* < .001, *partial* *η²* = .66) children improved in math fluency over time. The main effect of Age groups showed that older children had higher levels of math fluency at T1 (*t*(47) = -2.11, *p* = .04, *partial* *η²* = .10) and showed the same tendency at T2 (*t*(47) = -1.70, *p* = .09, *partial* *η²* = .06). More detailed information about the groups’ math fluency scores at each time point is provided in Table 1 and Figure 1B.
